# MST-AI: Skin Color Estimation in Skin Cancer Datasets

**DOI:** 10.3390/jimaging11070235

**Published:** 2025-07-13

**Authors:** Vahid Khalkhali, Hayan Lee, Joseph Nguyen, Sergio Zamora-Erazo, Camille Ragin, Abhishek Aphale, Alfonso Bellacosa, Ellis P. Monk, Saroj K. Biswas

**Affiliations:** 1 Electrical and Computer Engineering Department, Temple University, Philadelphia, PA 19122, USA; vahid.khalkhali@temple.edu (V.K.); saroj.biswas@temple.edu (S.K.B.); 2 Cancer Epigenetics Institute, Nuclear Dynamics and Cancer Program, Fox Chase Cancer Center, Philadelphia, PA 19111, USA; joseph.nguyen@fccc.edu (J.N.); sergio.zamora-erazo@fccc.edu (S.Z.-E.); alfonso.bellacosa@fccc.edu (A.B.); 3 Department of Medical Genetics and Molecular Biochemistry, Lewis Katz School of Medicine (LKSOM), Temple University Health System, Philadelphia, PA 19140, USA; 4 Department of Cancer and Cellular Biology, Lewis Katz School of Medicine (LKSOM), Temple University Health System, Philadelphia, PA 19140, USA; 5 Cancer Prevention and Control Program, Fox Chase Cancer Center, Philadelphia, PA 19111, USA; camille.ragin@fccc.edu; 6 Division of Dermatology, Melanoma and Skin Cancer Program, Fox Chase Cancer Center, Philadelphia, PA 19111, USA; abhishek.aphale@tuhs.temple.edu; 7 Department of Sociology, Harvard University, Cambridge, MA 02138, USA; emonk@fas.harvard.edu

**Keywords:** skin color detection, skin cancer detection, bias reduction, artificial intelligence (AI), Monk Skin Tone (MST) scale

## Abstract

The absence of skin color information in skin cancer datasets poses a significant challenge for accurate diagnosis using artificial intelligence models, particularly for non-white populations. In this paper, based on the Monk Skin Tone (MST) scale, which is less biased than the Fitzpatrick scale, we propose MST-AI, a novel method for detecting skin color in images of large datasets, such as the International Skin Imaging Collaboration (ISIC) archive. The approach includes automatic frame, lesion removal, and lesion segmentation using convolutional neural networks, and modeling normal skin tones with a Variational Bayesian Gaussian Mixture Model (VB-GMM). The distribution of skin color predictions was compared with MST scale probability distribution functions (PDFs) using the Kullback-Leibler Divergence (KLD) metric. Validation against manual annotations and comparison with K-means clustering of image and skin mean RGBs demonstrated the superior performance of the MST-AI, with Kendall’s Tau, Spearman’s Rho, and Normalized Discounted Cumulative Gain (NDGC) of 0.68, 0.69, and 1.00, respectively. This research lays the groundwork for developing unbiased AI models for early skin cancer diagnosis by addressing skin color imbalances in large datasets.

## 1. Introduction

Skin cancer is the fifth most prevalent form of cancer in the United States, with current estimates suggesting that one in five Americans will develop it in their lifetime [1,2]. Among the various types of skin cancer, melanoma is of particular concern due to its potential for metastasis and poor prognosis in certain populations. Although melanoma can affect individuals of all skin tones, its incidence is notably higher among white populations than among other racial and ethnic groups. For instance, the lifetime risk of developing melanoma is approximately 3% (1 in 33) for White individuals, compared to just 0.1% (1 in 1000) for Black individuals and 0.5% (1 in 200) for Hispanic individuals. Furthermore, melanoma incidence rates are 1.3 per 80,000 for Asian/Pacific Islanders and 10.7 per 80,000 for Native Americans [3,4,5,6].

Despite the lower incidence in individuals with darker skin tones, skin cancer, including melanoma, can still occur in these populations, and the prognosis is often worse due to delayed diagnosis [7]. Research has shown that patients with darker skin tones are more likely to be diagnosed at later stages of melanoma, making treatment more challenging and less effective [8]. In total, 12.6% of non-Hispanic Whites (NHWs) presented with regional or distant disease, compared to 21.0% of Hispanics and 34.1% of non-Hispanic Blacks (NHBs), who were diagnosed at more advanced stages [9]. In fact, 22% of melanoma cases in African−American patients are diagnosed when the cancer has spread to nearby lymph nodes, and 14% are diagnosed when it has metastasized to distant sites [10]. Moreover, individuals with darker skin tones are prone to melanoma in areas that are less commonly exposed to the sun, such as the palms of the hands, soles of the feet, groin, inside the mouth, and under the nails [11,12,13,14]. This disparity underscores the importance of improving early detection, awareness, and targeted interventions for skin cancer in all populations, regardless of skin type.

Recent research on skin cancer diagnosis has explored advanced imaging and AI techniques to improve detection across diverse skin types. Huang et al. [15] introduced a hyperspectral imaging approach combined with YOLOv5 [16] for skin cancer classification, demonstrating improved accuracy through detailed spectral information. Lin et al. [17,18] assessed the performance of the Spectrum-Aided Vision Enhancer (SAVE) system in detecting various melanoma subtypes, highlighting its potential utility across different lesion presentations. However, these studies primarily focused on technology efficacy without explicitly addressing the influence of skin color variability on diagnostic outcomes. Understanding and mitigating the impact of skin color is critical for developing equitable skin cancer detection tools that perform reliably across all populations.

Skin cancer detection from skin samples is a valid test for clinical diagnosis within the dermatology society. Unfortunately, many available medical datasets are not fair. This means that they usually have biases and tendencies toward one or some of the populations because of the geographical and temporal sources of data. These datasets are usually obtained from one, two, or three institutes in a single area or country with a fixed date range. Hence, it cannot be generalized to a wider range of patients. Moreover, patient data privacy enforces anonymization, and data such as race and ethnicity must be removed before distribution. This biased dataset affects the diagnosis process for medical students in training, as it was sourced from a single institution and contained a significant imbalance between data from White and Black patients [19]. In contrast, state-of-the-art artificial intelligence models are data-oriented, trained, validated, and deployed based on the available data. Unfairness in data creates unfair AI models. Unfair AI means that it can diagnose a disease for a specific population group better than other groups, such as skin cancer in white people compared to people of color [20,21].

To overcome skin color disparities, Daneshjou et al. [22] developed the publicly available, curated, and pathologically confirmed Stanford Diverse Dermatology Images (DDI) dataset. DDI dataset spans Fitzpatrick Skin Types (FST) I–VI [23,24] and was curated from Stanford Clinics between 2010 and 2020, and contains 656 images from 570 patients. It covers a wide range of skin tones, with 208 images from FST I–II, 241 from FST III–IV, and 207 images from FST V–VI. This allows for a direct comparison between lighter and darker skin tones while controlling for variables like age, sex, and diagnostic category. Although DDI is a valuable dataset, the number of samples is not sufficient to train and validate an AI system for clinical applications. Moreover, the Fitzpatrick Skin Type Scale is not an extensive indicator of human skin color [25,26,27].

To address the skin color disparity in skin cancer datasets, we leveraged the Monk skin tone (MST) scale [28,29], which offers reduced bias compared to the Fitzpatrick scale. We developed MST-AI models, a skin color estimation method based on the MST scale, and applied our models to the skin color of samples in a large publicly available skin cancer dataset, the International Skin Imaging Collaboration (ISIC) dataset. MST-AI automatically (1) removes image frames using a classical image processing algorithm, (2) segments and removes lesions using convolutional neural networks, and (3) models normal skin tones using a Variational Bayesian Gaussian Mixture Model (VB-GMM). We compared the resulting skin color distributions with the MST scale’s probability distribution functions (PDFs) using the Kullback-Leibler Divergence (KLD) metric. Validation was performed using manually annotated reference labels and comparisons with K-means clustering of image mean RGBs and skin mean RGBs. Our thorough validation demonstrates that MST-AI outperforms alternatives according to established ranking metrics such as Kendall’s Tau.

Bevan and Atapour-Abarghouei [28] made a valuable contribution by developing an automated skin tone detection method using the Fitzpatrick scale and applying bias unlearning techniques to enhance fairness in melanoma classification. Their approach did not include preprocessing steps, such as lesion or frame removal, which means that lesion and border colors could influence skin tone estimation. They also assigned a single skin tone per image, which may not fully capture the color variation due to overlaps within the Fitzpatrick scale. In comparison, our study uses the MST scale and incorporates preprocessing to focus on skin regions, offering an alternative perspective on automatic skin tone assessment in skin cancer diagnosis.

The remainder of this paper is organized as follows: Section 2 introduces the ISIC dataset and commonly used skin color scales and illustrates the proposed skin color estimator, detailing each step, including frame and lesion removal, the requirements for the skin color PDF, and the use of Kullback-Leibler Divergence (KLD). Section 3 presents the estimation results and compares them with the manual annotations. Section 4 provides a comparison and statistical analysis of the results. Finally, Section 5 concludes the paper with some comments and outlines directions for future work.

## 2. Materials and Methods

### 2.1. Dataset

The ISIC archive is one of the largest and most widely used repositories of dermoscopic images in melanoma research. With over 80,000 images, the collection encompasses a wide array of skin conditions, including benign and malignant lesions. Although all samples in the archive can be classified into two broad categories, benign and malignant, only a subset of the dataset is accompanied by detailed annotations. These annotations include lesion segmentation masks, which outline the precise boundaries of skin lesions, as well as essential metadata such as patient age, sex, and diagnosis [29]. The number of samples for the classification and segmentation tasks for each year is shown in Table 1 and Table 2, respectively.

We used the ISIC datasets spanning several years, which incorporated dermoscopic images collected from multiple international institutions. The multi-institutional and multi-year nature of the dataset ensured a broad representation of patient populations, imaging equipment, and clinical settings. The geographic diversity of the contributing centers introduces spatial variability, while the inclusion of data collected across different years adds temporal diversity. This combination enhances the heterogeneity of the dataset, supporting the development and evaluation of models that are more likely to generalize well across diverse, real-world conditions. Consequently, the use of the ISIC dataset provides a strong foundation for building robust and widely applicable algorithms, reducing the risk of dataset bias and overfitting to specific clinical settings. This rich combination of annotated images and metadata makes the ISIC archive an invaluable resource for a variety of research and clinical applications. It supports tasks such as (1) melanoma detection, where machine learning algorithms can be trained to distinguish between benign and malignant lesions, (2) lesion segmentation, which involves identifying and isolating skin lesions in images, and (3) diagnostic predictions, where predictive models can utilize patient metadata and image data to forecast the likelihood of various skin conditions. Thus, the breadth and depth of the dataset make it a versatile and essential tool for advancing melanoma research and improving diagnostic accuracy in dermatology [30,31,32].

Although the ISIC datasets contain many samples, which is one of the largest publicly available datasets, they lack information on race, ethnicity, and skin color groups or labels, which can lead to inaccurate predictions of skin melanoma. This research investigates the development of an AI-based method for estimating skin color from skin lesion image samples.

### 2.2. Skin Color Scales

Two well-known scales for skin color or tone are the Fitzpatrick [23] and Monk [33] skin tone (MST) scales. Both the Fitzpatrick and Monk scales assess skin color types but serve different purposes and have distinct methodologies. The Fitzpatrick scale, introduced by Dr. Thomas Fitzpatrick in 1975 [23], categorizes skin into six types based on its response to UV radiation, particularly focusing on whether the skin tends to burn or tan. These categories range from Type I, which always burns and never tans, to Type VI, which rarely burns and always tans. This scale is widely used in dermatology to evaluate the risk of sun damage, the likelihood of developing skin cancer, and to determine appropriate sun protection strategies for different individuals [23,24].

**Figure 1:**
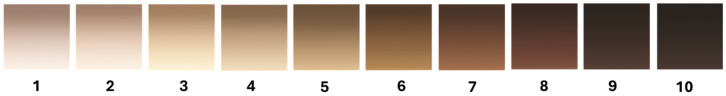
Monk skin tone (MST) scale [34].

The Monk skin tone (MST) scale, developed by Dr. Ellis P Monk [33], takes a broader approach to classifying skin tone types. It is designed to be more generalizable across different populations and regions, as it focuses on a wider range of skin characteristics that are less influenced by UV exposure. The MST scale seeks to categorize skin color groups based on broader environmental and genetic factors, making it more versatile across varying climates and ethnicities, as shown in Figure 1. It is not specifically tied to sunburn or tanning but looks at overall skin characteristics that can vary more widely between individuals. While the Fitzpatrick scale is primarily concerned with sun-related skin reactions, the MST scale offers a more holistic view that can be applied across diverse demographics, providing insights into skin sensitivity and adaptability beyond just UV response [25,26,27].

### 2.3. Skin Color Estimator

**Figure 2:**
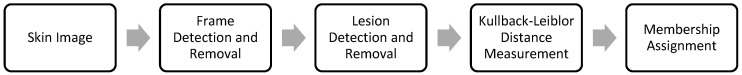
Schematic of the skin color classifier model.

An ISIC sample can be divided into three regions: the frame, lesion, and normal skin. The frame is typically a dark (or occasionally white) area surrounding the image, while the lesion is the suspicious region, which may be benign or malignant. Although not all images in the dataset contain these regions, normal skin usually remains after the removal of the frame and lesion. Given that the colors of both the frame and lesion differ significantly from those of normal skin, it is crucial to detect these regions to accurately estimate the skin color. We developed a model to estimate the skin tone of samples in the ISIC dataset based on the MST scale. A schematic of the proposed model is shown in Figure 2. The source code of the proposed system is publicly available at the GitHub repository (https://github.com/comp-epi-lee-lab/skin_color_estimator; released version: v1.0.0; accessed on 12 July 2025). Each step is described in detail below.

#### 2.3.1. Frame Detection and Removal

**Figure 3:**
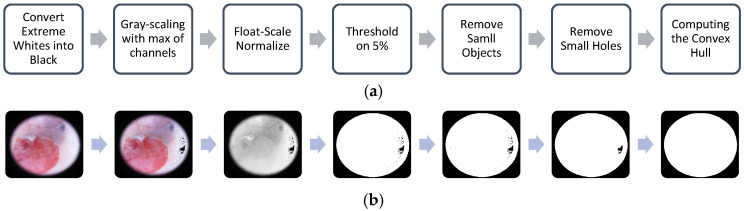
(**a**) Flowchart of frame segmentation steps; (**b**) Example images for each step of the frame removal algorithm: steps 1–7 in Algorithm 1.

Manually annotating medical data is time-consuming, erroneous, and labor-intensive, making unsupervised methods an attractive alternative for saving time and money. Certain characteristics can aid in developing algorithms for frame detection. Frames typically appear as solid black or white areas at the four corners of the sample images. Based on these traits, we created an algorithm to detect the frames. The algorithm is outlined in Algorithm 1, and a flowchart of the algorithm is shown in Figure 3a.

Similar to other unsupervised algorithms, certain parameters in this frame detection method must be manually or heuristically determined and tuned according to the dataset. The input to the algorithm is a color image in RGB format, and the output is a binary mask where ‘1’ indicates the frame region. The four key parameters, namely, white, black, object, and hole thresholds, were heuristically set to 95%, 5%, 64 pixels, and 64 pixels, respectively. The white and black thresholds define the upper and lower percentiles of the colors for non-frame areas. The object and hole thresholds specify the minimum size (in pixels) for the frame within the image and for the non-frame regions within the frame, respectively. After defining these thresholds, the algorithm begins by converting the extreme white regions into extreme black regions (Step 1). Next, the grayscale image is analyzed by taking the maximum value of the RGB channels, rather than their mean value, to enhance the contrast (Step 2). This approach functions similarly to a high-pass filter because it highlights high-contrast areas while filtering out low-contrast regions. The grayscale image is then normalized to the range [0, 1] (Step 3) and binarized using the black threshold (Step 4). The output mask from this step highlights the extremely dark regions, which are expected to correspond to the frame, although very dark spots (e.g., lesions) within the image may also resemble the frame color. Therefore, both the frame and dark spots are detected in Step 4. However, the frame area is not entirely dark, and some brighter spots within the frame region may remain undetected. These bright spots inside the frame are classified as “holes,” while the dark spots are referred to as “objects.” Step 5, which is divided into three sub-steps, removes small objects that are likely not related to the frame. The algorithm identifies objects using eight-neighbor connectivity, computes the area of each object by summing the binary pixel values, and removes objects with an area smaller than the object threshold. Step 6 mirrors Step 5 but operates on a negative binary image. To improve the estimation of the skin region (including both normal skin and lesions), a convex hull estimation [35] is applied (Step 7). The final output of the algorithm is a region of interest (ROI) representing the skin region, which includes normal skin and lesion areas. The frame detection algorithm consists of seven steps, with the outputs from each step shown in Figure 3b for an example image from the ISIC dataset.
**Algorithm 1**: Frame segmentation and removal.**Input**Three color channels (red, green, blue) of input image: I = <R, G, B>Each R, G, and B channel is a matrix with r rows and c columns.**Output**The binary mask of the frame**Initialization**The white threshold is manually determined by $W_{th}$ = 0.95The black threshold is manually determined by $B_{th}$ = 0.05The object threshold is manually determined by $O_{th}$ = 64The hole threshold is manually determined by $H_{th}$ = 64**Steps**Convert extreme whites into black$$I_{1}=\left\{\begin{array}{c} I,\;\;\;if\;\frac{1}{3}\left(R+G+B\right)<{256\times W}_{th}\\ 0,\;\;\;if\;\frac{1}{3}\left(R+G+B\right)\geq {256\times W}_{th}\; \end{array}\right.$$Gray scale version of the image is computed as
$$I_{2}=\max\left(R,\;G,\;B\right)$$Float scale normalization of the gray scale image is computed as$$I_{3}=\frac{\left(I_{2}-{I_{2}}_{min}\;\right)}{\left({I_{2}}_{max}-{I_{2}}_{min}\right)}$$Here matrix division is an element-wise operation.The black regions are separated by manual thresholding. The output is a binary matrix.$$I_{4}=\left\{\begin{array}{c} 0,\;\;if\;I_{3}<B_{th}\\ 1,\;\;if\;I_{3}\geq B_{th}\; \end{array}\right.$$Small objects are removed5.1.Label each region by finding the eight-neighbor connectivity in all pixels. Each region is assumed to be an object. Object labels are $0,\;1,\;2,\;\ldots ,\;n_{obj}-1$ which $n_{obj}$ is the total number of objects (regions) on the binary image.5.2.Objects areas are computed by$$A_{k}=\sum_{i=0}^{r-1}\sum_{j=0}^{c-1}\left\{\begin{array}{c} 1,\;\;p_{ij}=k\\ 0,\;\;oth \end{array}\right.\;,\;\;\;\;\;\;\;\;\;\;\;\;\;k\in 0,\;1,\;2,\;\ldots ,\;n_{obj}-1$$ where $p_{ij}$ is the pixel value at $\left(i,\;j\right)$ in a binary image $I_{4}$.5.3.Objects smaller than the given threshold are converted to background (removed):$$I_{5}=\sum_{k=0}^{n_{obj}-1}\left\{\begin{array}{c} 0,\;\;if\;A_{k}<O_{th}\\ 1,\;\;if\;A_{k}\geq O_{th}\; \end{array}\right.$$Small holes are removed. The algorithm is exactly similar to step 5, but instead of labeling pixels with value 1, pixels with value 0 will be labeled. Also, the threshold for holes $H_{th}$ is used.Convex Hull is computed for smooth segmentation. The convex hull is the set of pixels included in the smallest convex polygon that surrounds all white pixels in the input image. The area inside this convex hull will be considered as the region of interest (ROI) and anything outside as the frame.

#### 2.3.2. Lesion Segmentation

Following frame detection, it is essential to identify and segment the lesion region, regardless of whether it is benign or malignant. This is because the color of the lesion typically differs significantly from that of normal skin, and including it in the skin color estimation would lead to substantial errors.

In contrast to frame detection, unsupervised lesion detection is highly challenging due to the wide variety of patterns and shapes that lesions can exhibit. Fortunately, subsets of the ISIC dataset from 2016, 2017, and 2018 included lesion masks. We used these subsets to develop a fully convolutional neural network (FCNN) model for the automatic semantic segmentation of lesions [36]. A transfer learning approach on PyTorch/v2.3.1 [37] pre-trained FCN-50 [36] is re-trained on training datasets of ISIC 2016 to 2018 and tested on their test cohorts. The images are batched into 32 samples and augmented with random horizontal/vertical flips, random translation, rotation, scale, shearing, and perspective, and then resized to 256 × 256 pixels and normalized to [0, 1]. Adam optimizer with a learning rate of $10^{-4}$ tuned the model to the data in 20 epochs. Jaccard loss is minimized as the optimization metric with the following definition:

(1) $$Jaccard\;Loss=-log(\frac{\sum_{c}\sum_{r}X.Y}{\sum_{c}\sum_{r}X+\sum_{c}\sum_{r}Y-\sum_{c}\sum_{r}X.Y})$$

where $X,Y,\Sigma_{c}$, and $\Sigma_{r}$ are actual binary mask, detected mask (after sigmoid), element-wise product, summation over columns, and summation over rows, respectively. Jaccard Loss is an extension to the intersection over union match of two binary images that is rescaled to a logarithmic range for better convergence with stochastic gradient optimizers, such as Adam.

The training and testing results are presented in Table 3. In addition to Jaccard Loss, the DICE score is included as a well-known metric for better comparison. DICE score is defined as:

(2) $$DICE=\frac{2\sum_{c}\sum_{r}X.Y}{\sum_{c}\sum_{r}X+\sum_{c}\sum_{r}Y}$$

**Figure 4:**
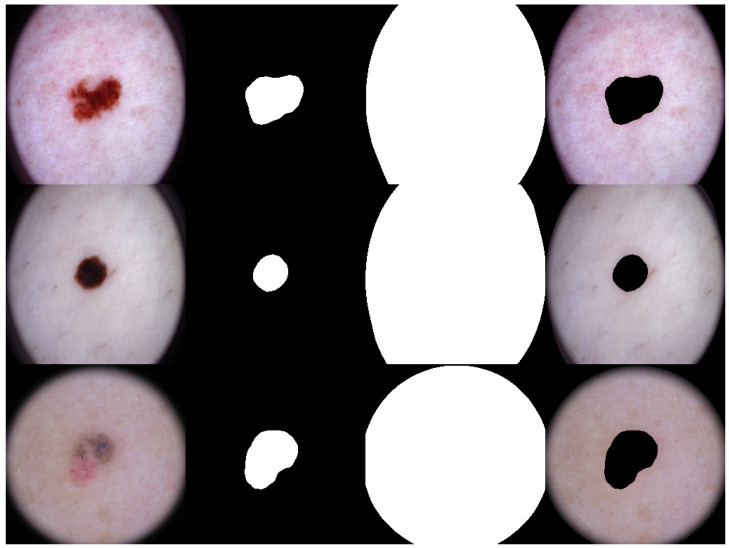
Examples of Normal Skin Detection, from **left** to **right**: original image, detected lesion, detected frame, and remaining normal skin.

The high DICE score for lesion detection in unseen test data confirmed that the FCN-50 model effectively excluded a substantial portion of the lesion, allowing the remaining areas to be used for color estimation of normal skin. Three examples of normal skin segmentation are shown in Figure 4. It is important to note that the frame and lesion removal steps can be performed in either order, as they are independent of one another.

#### 2.3.3. Color Density Estimation

**Figure 5:**
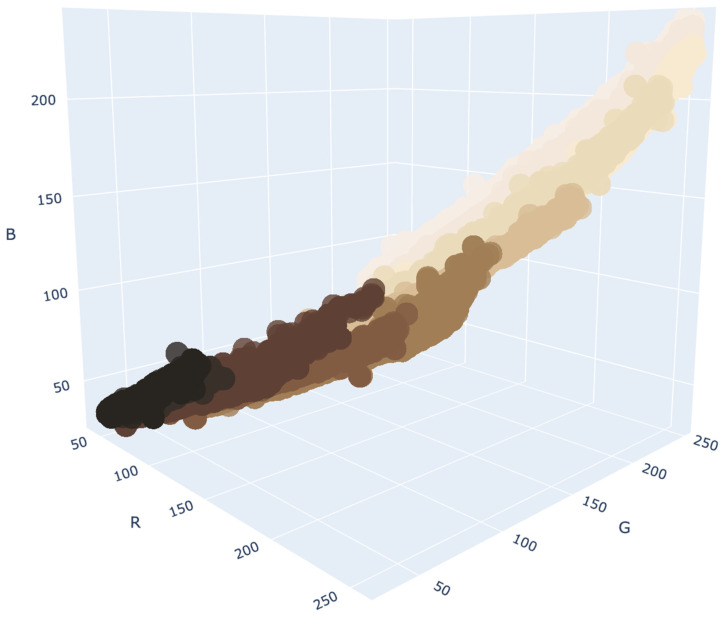
MST scales in 3D RGB; each color corresponds to one of the MST scales shown in Figure 1.

The central question is whether it is possible to assign a single MST scale value to the skin color in dermoscopic images. To address this, we can examine the MST scales in a 3D plot, as shown in Figure 5. This plot is generated by normalizing the RGB values of the MST scale depicted in Figure 1. As observed in Figure 5, the MST scale exhibits a significant overlap, indicating that neither an MST nor a skin color can be represented by a single RGB point in space. While the MST scales are associated with a primary color that varies in shade, this is not the case for skin. Normal skin may consist of several base colors combined with different tones. Therefore, both the MST and skin color represent a distribution of points (pixels) in the 3D RGB space. This distribution can be modeled using a PDF.

Probability density functions (PDFs) can be estimated using various methods, with the kernel method being one of the most commonly used approaches [38]. This method assumes that the distribution can be approximated using a weighted sum of kernels. The Gaussian kernel is the most frequently used due to its ability to satisfy key kernel conditions, including non-negativity, symmetry, and smoothness.

Visual examination of the 3D plots of the color distributions suggests that a single Gaussian distribution is insufficient for accurately estimating the skin color PDF. However, a more precise representation can be obtained using multiple Gaussians or a Gaussian Mixture Model (GMM). The GMM is a parametric approach that utilizes the kernel method to estimate the density function. It is assumed that the data are generated from a mixture of several Gaussian distributions, with each component characterized by its own mean and variance (or covariance in the multivariate case), as shown in the following equation [38]:

(3) $$f\left(x\right)=\sum_{k=1}^{K}w_{k}N\left(x|\mu_{k},\Sigma_{k}\right)$$

where K, $w_{k}$, and $N\left(x|\mu_{k},\Sigma_{k}\right)$ are the number of components, the weight of the *k*-th component, and the Gaussian distribution with a mean of $\mu_{k}$ and covariance of $\Sigma_{k}$, respectively.

**Figure 6:**
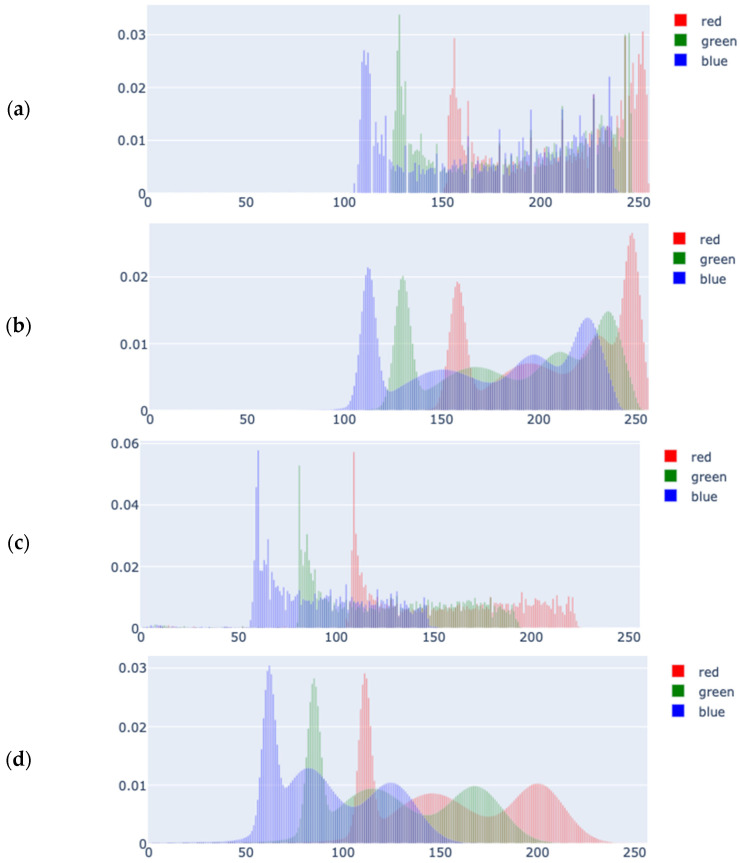
The estimation of two MSTs with VB-GMM; (**a**) MST 1 samples, (**b**) VB-GMM estimation of MST 1, (**c**) MST 5 samples, (**d**) VB-GMM estimation of MST 5.

Although GMM is an effective method for density function estimation, it carries the risk of overfitting or underfitting because the number of components must be predetermined. A more robust alternative is the Variational Bayesian Gaussian Mixture Model (VB-GMM), which addresses these concerns. VB-GMM treats model parameters as random variables, assigns prior distributions to them, and aims to compute a posterior distribution over the parameters rather than just providing point estimates. The regularization introduced in the VB-GMM reduces the need to fix the exact number of components. In our approach, we used eight components to estimate the PDFs. Although we observed that half of these components overlapped with others, the computational overhead was an acceptable trade-off for minimizing underfitting and estimation errors. Two examples are shown in Figure 6 in 2D (each RGB component separately) for better visualization, as they are originally in 3D (RGB).

Our approach fits a separate VB-GMM to each of the 1618 image samples individually, estimating the RGB probability distribution in the 3D color space per sample. Because each model is computed independently, the global imbalance of skin tones in the dataset does not influence the performance of the VB-GMM.

After estimating the PDF, we generated random samples with similar coordinates (RGB values) to measure the differences and similarities in the next step. The red, green, and blue values were integers ranging from 0 to 255. We normalized these values to a range between 0 and 1, knowing that there are only 256 possible states for each variable. Consequently, we expect that a similar spatial sampling rate will be sufficient for an accurate PDF estimation. In practice, we selected 100 samples per axis, resulting in a total of 100^3^ = 1,000,000 points. We found that increasing the sample count to 256^3^ = 16,777,216 did not yield significant improvements in the results, but it was computationally much more expensive.

#### 2.3.4. Kullback-Leibler Divergence and Membership Scores

In the previous section, we have estimated the PDFs of all MST scales and the skin sample. We now want to determine how similar or different the two PDFs are. A well-known metric is Kullback-Leibler divergence (KLD), defined as

(4) $$D_{KL}\left(P∥Q\right)=\sum_{x\in \chi }P\left(x\right)\log\left(\frac{P\left(x\right)}{Q\left(x\right)}\right)$$

where $P\left(x\right)$ and $Q\left(x\right)$ are actual and detected PDFs, respectively, which are defined on the domain χ. $D_{KL}\left(P∥Q\right)$ measures the information loss by estimating the $P\left(x\right)$ by $Q\left(x\right)$. If we assign a skin color to a MST scale in an image, it must represent the MST scale in the best possible form. It means that the skin PDF must represent the MST PDF, so $P\left(x\right)$ is the MST PDF and $Q\left(x\right)$ is the skin color PDF.

Since all MST scale PDFs and a skin color sample have overlap theoretically, ten KLDs are calculated per skin image sample. These KLDs (or other distance metrics such as Euclidean distance) are deployed to define the membership score, as shown below:

(5) $$M\left(P,Q_{m}\right)=softmax\left(1-\frac{D_{KL}\left(P∥Q_{m}\right)}{\underset{m}{\max}D_{KL}\left(P∥Q_{m}\right)}\right)$$

where P and $Q_{m}$ are PDFs of the skin color and m-th MST scale, respectively. The softmax function is defined as

(6) $$softmax\left(x_{m}\right)=\frac{e^{x_{m}}}{\sum_{m}e^{x_{m}}}$$

$M\left(P,Q_{m}\right)$ is a normalized score between [0,1], where higher values indicate higher similarity.

Both GMM and KLD involve operations that are computationally sensitive to underflows and overflows, as GMM is exponential, and KLD includes a logarithmic term. Using standard floating-point calculations, such as float32 or float64, can lead to frequent computational failures when calculating the KLD, rendering the membership score computation infeasible. To address this, we developed a computationally efficient method that combines the KLD and GMM to prevent underflows and overflows. This method is described in Appendix A.

### 2.4. Test Cohort Annotation

To assess the validity of our membership definition in the ISIC datasets, we used human annotation as the gold standard. Our human annotator was tasked with segmenting an area of normal skin by drawing a rectangle around it, with everything inside the rectangle representing the expected skin color. We validated our estimator by calculating the statistical correlation between the manually segmented areas and the skin color estimator. For each image sample, we obtained ten membership measurements for each MST scale and annotated area, as well as ten membership measurements for each MST scale, and the skin area was automatically generated by our model in a semi-supervised manner. We then computed the ranked correlations between these ten values to evaluate how closely our model aligns with human judgment.

**Figure 7:**
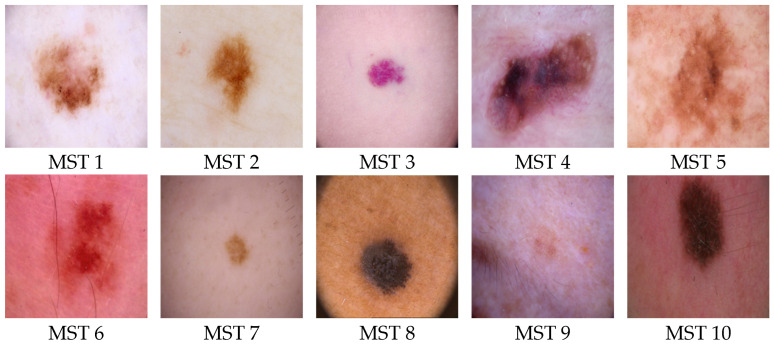
Examples of MST scale pre-classification for the annotation process.

The ISIC dataset contains approximately 80,000 images, making it impractical to have an annotator manually label all of them. To address this, we randomly selected a subset of images that adequately represented color diversity. While random selection typically reflects the sample prevalence in the dataset, we recognize that the ISIC datasets inherently exhibit an imbalance in skin color and fairness. To mitigate this issue, we pre-classified skin color using an automatic approach. Although this pre-classification is not highly precise, it is sufficient for a weak categorization of skin colors across the MST scale. In this weak classifier, the mean RGB values of all pixels in the segmented normal skin region were used to represent the skin color. Similarly, the MST scale representation points were determined using the mean RGB values of all pixels in the MST scale. A nearest-neighbor approach based on the Euclidean distance between the RGB means was then used to assign a skin color label. We pre-classified the skin colors into ten MST scales and randomly selected 200 images from each scale, resulting in 2000 images prepared for manual annotation. Examples are shown in Figure 7.

**Figure 8:**
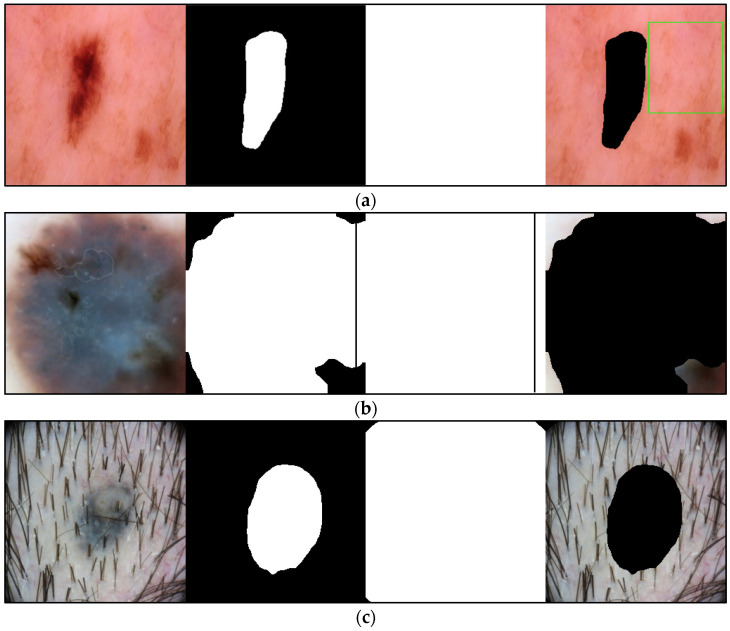
Three examples of the included and excluded images. (**a**) Included image from left to right: original image, detected lesion, detected frame, normal skin; (**b**) Excluded due to insufficient skin area; (**c**) Excluded due to hair. The green rectangular box shows the manual annotation by the human annotator.

During the annotation process, we identified two categories of images that should be excluded from the computation: those in which the normal skin area is too small to annotate and those in which body hair or other artifacts obscure almost the entire normal skin region. To address this, we divided the images in each MST scale into two groups: “included,” which contains manually annotated images, and “excluded,” which does not contain annotations. Figure 8 shows examples of these annotations. Of the 2000 images, 1618 were included for annotation, while 382 were excluded.

Although the pre-classifier was designed to create a weakly balanced dataset by selecting an approximately equal number of samples across different MST scales, we observed a higher exclusion rate of images in darker MST categories. This was primarily due to the presence of image artifacts that disproportionately affected the samples.

Addressing this imbalance requires a human-in-the-loop annotation pipeline to manually curate and correct the dataset, an approach that is both costly and time-intensive. Given these constraints, we acknowledge the remaining imbalances in the dataset. However, it represents a substantial improvement over the original ISIC dataset, which exhibited a more pronounced imbalance across skin tone categories.

## 3. Results

Three skin color categorization methods were compared using 1616 image samples. The methods are Image K-means, Skin K-means, and MST-AI, the proposed skin color estimator.

In both the Image K-means and Skin K-means methodologies, each sample is represented as a point in a three-dimensional RGB color space, where the coordinates correspond to the mean red, green, and blue intensity values of the associated pixel set. In the case of Image K-means, this pixel set comprises all pixels within the image, including those belonging to the frame and lesion regions. Conversely, Skin K-means uses only the pixels identified within the skin region, explicitly excluding both the frame and lesion pixels. Identical RGB-based coordinate representations are also assigned to each reference color in the MST scale [34]. For each sample, the Euclidean distance to every MST scale reference coordinate is computed and subsequently employed as the distance metric for determining the membership scores, as defined in Equation (5).

In the Skin Color Estimator framework, the dissimilarity between the samples and MST scale references is quantified using KLD. Specifically, the KLD is calculated between the VB-GMM estimated PDF of the sample and those of the MST scale reference distribution. These KLD values are then used to derive the membership scores in accordance with the formulation presented in Equation (5).

Table 4 indicates the ranks of the MST scales, ordered from the best to the worst match (only the top four out of ten values are shown for brevity). As shown, MST-AI provides the closest and most comprehensive match to the annotated regions. Although the other two methods perform relatively well for lighter skin tones, they struggle to accurately capture the diversity of darker skin tones. This limitation arises because these methods tend to make assumptions based on fewer characteristics or a narrower range of skin tones, which causes them to underperform when the skin color is darker or more complex. In these cases, Image and Skin K-means are particularly challenged when dealing with skin that contains multiple tones or variations in MST scales. These methods are less effective in identifying the subtleties of such diversity, resulting in less accurate matches for complex skin colors.

In contrast, MST-AI adopts a broader approach to classification by considering a wider selection of possible matches across different MST scales. This allows MST-AI to be more adaptable and better suited to handle a variety of skin tones, including those with greater complexity or diversity. This flexibility is particularly important in the decision-making process, as it allows the MST-AI to rely not only on the top-ranked match but also to take into account the second, third, or even fourth-ranked options if the top-ranked match does not fit well. This diversity in selection makes the MST-AI more robust, as alternative options can still offer a good fit, improving the overall reliability of the results.

In comparison, the other two methods do not exhibit the same flexibility. Their second, third-, and fourth-ranked matches are essentially extensions of the top-ranked match, meaning that they are still heavily influenced by the first selection. If the top-ranked match is incorrect or suboptimal, the subsequent options are less likely to provide a better alternative. This lack of diversity in the ranking process implies that the reliability of these methods is compromised if the top-ranked match is not ideal.

For further analysis, we employed two procedures to quantitatively evaluate the ranking results. To conduct a thorough evaluation, we began by plotting a histogram that represents the distribution of rankings for the top-1 match across all three methods. This histogram was compared to the annotation benchmark, which serves as the gold-standard manual annotation and is visually presented in Figure 4. The goal of this analysis is to assess how well the rankings of each method align with the reference benchmark across the various MST scale categories.

The top-1 ranking histogram shows the distribution of the highest-ranked match for each method. By comparing the histogram with the benchmark, we are able to assess how closely the rankings of each method resemble the distribution of samples observed in the manual annotations.

**Figure 9:**
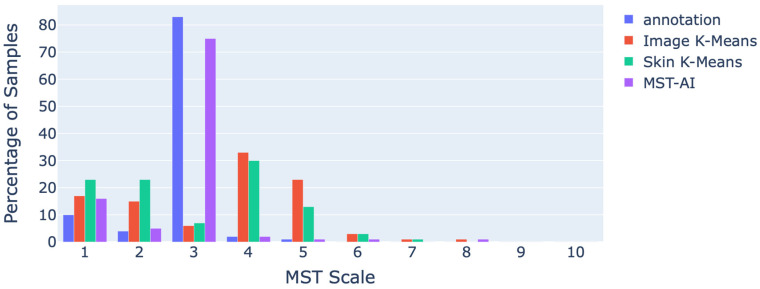
The top-1 rankings.

From the histograms in Figure 9, it is evident that the MST-AI stands out as the most accurate in closely matching the distribution of the gold-standard manual annotations across all MST scale bins. This indicates that the MST-AI can consistently estimate the skin tone distributions represented in the annotated dataset, whether the skin tones fall in the lighter or darker MST scale categories. The closeness of the histograms demonstrates that the KLD of PDFs is more in tune with the actual skin tone distributions, effectively capturing the variation in skin tones as represented by the annotated data. In contrast, the other two methods, although they may perform well in specific areas (e.g., lighter skin tones), show discrepancies when compared with manual annotations, particularly in terms of how they distribute the top-1 ranking. The ability of the MST-AI to closely align with the benchmark across all MST scale bins highlights its superior performance in reflecting the real-world diversity of skin tones in a more comprehensive manner.

Next, we investigated the comparison of individual sample rankings using ranking metrics. There are several well-known metrics to measure ranking performance, such as Precision at K, Recall at K, Kendall’s tau [39,40], Spearman’s Rank Correlation [41], and Position-based Accuracy. Although Precision and Recall at K show the performance of the estimator, they do not distinguish the order of ranks in the top-K results. Because the order of MST scale is important in our estimator, we use Kendall’s Tau, Spearman’s Rank Correlation, and Normalized Discounted Cumulative Gain (NDCG), which are all sensitive to rank orders.

Kendall’s tau is defined as:

(7) $$\tau =\frac{\sum_{i=1}^{k}\sum_{j=i+1}^{k}\left\{\begin{array}{cc} 1, & \mathrm{if}\;\left(A_{i}-A_{j}\right)\left(D_{i}-D_{j}\right)>0\\ -1, & \mathrm{otherwise} \end{array}\right.}{\left(\begin{array}{c} k\\ 2 \end{array}\right)}$$

where $A_{i}$, $D_{i}$, and k are the actual rank, detected rank, and the number of top results, respectively.

Spearman’s Rank Correlation is defined as

(8) $$r_{s}=1-\frac{6\sum_{i=1}^{k}d_{i}^{2}}{k\left(k^{2}-1\right)};\;where\;d_{i}=A_{i}-D_{i}$$

where, similar to Kendall’s Tau equation, $A_{i}$, $D_{i}$, and k are the actual rank, detected rank, and the number of top results, respectively. This equation is valid as long as there are no ties in the ranking, which is almost correct since the probability of ties is rare (if not zero) in our calculations, which can be ignored.

NDCG is defined as

(9) $$NDCG=1-\frac{\sum_{i=1}^{k}\frac{2^{{md}_{i}}-1}{\log_{2}\left(i+1\right)}}{\sum_{i=1}^{k}\frac{2^{{ma}_{i}}-1}{\log_{2}\left(i+1\right)}}$$

where ${ma}_{i}$ and ${md}_{i}$ are actual and detected membership scores for top-K results, respectively.

The Kendall’s Tau and Spearman’s Rank Correlation scores are between −1 and +1, where −1 indicates an extreme difference and +1 indicates complete similarity. NDCG score varies between 0 (dissimilar) and 1 (similar). The performance evaluation results are presented in Table 5.

## 4. Discussion

### 4.1. Statistical Analysis of the Results

Because Kendall’s Tau and Spearman’s correlation produce very similar results across individual models, we report only one for comparison. A two-tailed Student’s *t*-test was used to assess the statistical significance of the mean results. Both Kendall’s Tau and Spearman’s Correlation are treated as random uniform variables ranging between a=−1 and a=+1. Standard deviation of these samples would be as

(10) $$\sigma =\sqrt{\frac{{\left(b-a\right)}^{2}}{12}}\approx 0.5774$$

with similar calculations on NDCGs, which are between [0, +1], the standard deviation would be 0.2887.

The *p*-values for each pair of mean results were computed using a two-tailed Student’s *t*-test. Based on the conventional *p*-value of 5% in most medical applications, if the *p*-value is less than 0.05, then the difference between the two results is statistically significant, and we can assume that the method with the higher mean value is significantly better than the method with the lower mean value.

The *p*-values are presented in Table 6. The circles represent the methods listed in Table 5, the dashed line depicts the statistical difference, and the continuous line implies that the difference is not statistically significant.

For the top-1 results (first row in Table 6), correlation metrics cannot be computed due to their inherent definitions; however, NDCG scores are reported. Although the NDCG values in Table 5 appear close to each other, the differences between the methods are statistically significant (*p* < 0.01), likely due to the moderately large sample size (e.g., 1600 samples).

For the top-2 (second row in Table 6), the correlation scores between methods 1 and 2 are not significantly different. However, method 3 demonstrated a significant improvement over both methods 1 and 2, with a *p*-value less than 0.01. Additionally, when evaluating the NDCG scores at the top-2, all three methods showed significant differences in performance.

In the case of top-3 (third row in Table 6), the correlation scores between methods 1 and 2 are significantly different. However, the NDCG scores for the two methods do not show a significant difference. Regardless, method 3 consistently outperforms both methods 1 and 2 in both the correlation and NDCG metrics.

For top-4 (last row in Table 6), method 3 significantly outperforms both methods 1 and 2, but there is no significant difference between methods 1 and 2 in either metric (correlation or NDCG).

The statistical differences reported in Table 5 and Table 6 indicate that Kendall’s τ and Spearman’s correlation are more sensitive to changes in the ranking order of outputs than NDCG, which appears more tolerant to such variations. This suggests that rank-based correlation metrics may better capture subtle shifts in the prediction order, especially when evaluating consistency across methods.

Additionally, the results show that Skin K-Means outperforms Image K-Means in three of the seven evaluation metrics, emphasizing the effectiveness of frame and lesion removal in accurately identifying normal skin regions. This highlights the value of incorporating preprocessing steps tailored to address domain-specific challenges. Notably, MST-AI, our proposed method achieves a consistently superior performance, showing statistically significant improvements over both baseline methods, Image K-means and Skin K-means, across all three metrics. This demonstrates the robustness and overall advantage of our approach for this task.

### 4.2. Caveats and Limitations

We randomly sampled data from multiple years of the ISIC dataset. The test cohort comprised 2000 manually annotated samples, providing sufficient statistical power for reliable estimation with appropriate confidence levels. While this manually annotated dataset significantly contributes to our results, it also introduces several limitations, some inherent to the dataset and others that could be addressed in future research. Below, we outline these considerations.

First, the dataset lacks metadata on imaging conditions, including the specific instruments used and background lighting. As a result, there is no gold standard or reference available to evaluate how effectively any background illumination detection method (if present) compensates for the effects of instrumentation or lighting variations. This absence limits our ability to assess or correct for confounding variables.

Second, skin color can vary substantially across different regions of an individual’s body. Therefore, assigning a single skin color to a person based on a small, region-unknown sample may not be reliable.

Third, due to the cost of manual annotation, we relied on a single set of annotations. This process is inherently subjective, similar to most human annotations. Different annotators may produce different labels for the same image. Inter-rater agreement is a commonly used metric in medical image analysis for quantifying annotation subjectivity. Although our dataset is publicly available, we plan to expand it in the future to include annotations from multiple raters, enabling the calculation of inter-rater agreement scores.

In summary, while our method and annotated dataset aim to provide a robust unsupervised approach for skin color estimation using ISIC samples, they are constrained by the lack of information about the body region, environmental illumination, and imaging instrumentation. Without access to such real-world metadata, further improvements in reliability and validation are challenging.

## 5. Conclusions

Publicly available skin cancer datasets often lack skin color information, which is crucial for the diagnosis and prognosis of skin cancer. Our study explored various skin color scales and found that the MST scale offers a less biased alternative to the well-established Fitzpatrick scale, particularly in non-white populations, such as individuals with darker skin tones. Based on the MST scale, we proposed a novel skin color detection method that leverages large, publicly available skin cancer datasets, such as the ISIC archive.

In this research, we addressed key challenges, including the removal of image frames and lesions, by developing an unsupervised automatic frame detection and removal technique. We then used the cleaned data to construct a robust convolutional deep neural network for lesion segmentation. Once the frame and lesion were removed, the remaining normal skin was analyzed for its various colors, tones, and shades. We modeled the normal skin pixels as a PDF using the Variational Bayesian Gaussian Mixture Model (VB-GMM), which is effective in capturing the underlying data distribution and is less sensitive to the predefined number of clusters.

We compared the PDF of normal skin with those of the MST scale using the Kullback-Leibler Divergence (KLD) as a metric to quantify the similarity between the distributions. To address KLD’s sensitivity to computational under-/overflows, we developed an efficient approximation method to mitigate these issues.

Finally, we compared our proposed method with the K-means clustering approach applied to both whole images and skin regions. The results, validated against manual annotations of approximately 1600 images, showed that our method outperformed others in matching MST scale classifications to normal skin samples, as evidenced by Kendall’s Tau, Spearman Correlation, and NDCG metrics.

As a next step, we aim to extend this work to develop more equitable AI-based systems for skin cancer detection. By addressing skin tone imbalances present in large-scale dermatology datasets, our method provides a foundation for fairer and more accurate diagnostic tools. Future work will focus on integrating advanced deep learning models to enhance skin cancer detection across all skin tone scales, with the goal of improving diagnostic performance and reducing bias in real-world clinical applications.

## Figures and Tables

**Table 1:** Classification task: number of samples and percentage of malignancy (vs benign) for different years in the ISIC datasets.

Year	Train	Malignant%	Valid	Malignant%	Test	Malignant%	Total	Malignant%
**2016**	900	19.22	0	0.00	379	19.79	1279	19.39
**2017**	2000	18.70	150	20.00	600	19.50	2750	18.95
**2018**	10,015	19.51	193	22.80	1512	20.30	11,720	19.67
**2019**	25,331	36.87	0	0.00	N/A	N/A	25,331	36.87
**2020**	33,126	1.76	0	0.00	N/A	N/A	33,126	1.76

**Table 2:** Segmentation task: number of samples and percentage of malignancy (vs. benign) for different years in the ISIC datasets (#segmented images/#total images).

Year	Train	Valid	Test	Total
**2016**	900/900	0	379/379	1279/1279
**2017**	2000/2000	150/150	600/600	2750/2750
**2018**	2594/10,015	100/193	1000/1512	3694/11,720
**2019**	0	0	0	0
**2020**	0	0	0	0

**Table 3:** Results of training and testing of FCN-50 on the ISIC 2016–2018 datasets.

	Train	Validation	Test
Jaccard Loss	0.0046	0.0071	0.0075
DICE	94.07%	89.50%	89.39%

**Table 4:** Three examples of MST scale assignments for each method.

Original Image	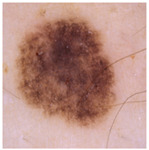	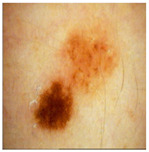	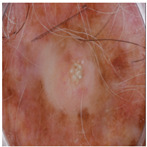
Annotated Region	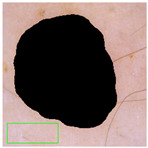	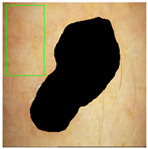	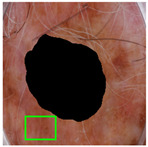
Image K-Means MST scale Memberships	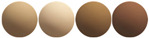 5,4,6,7 0.151, 0.128, 0.113, 0.101	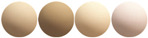 4,5,3,1 0.143, 0.137, 0.114, 0.108	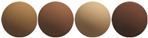 6,7,5,8 0.152, 0.138, 0.128, 0.113
Skin K-Means MST scale Memberships	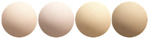 2,1,3,4 0.140, 0.138, 0.136, 0.120	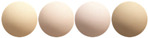 3,2,1,4 0.140, 0.140, 0.136, 0.124	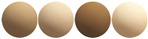 5,4,6,3 0.145, 0.128, 0.114, 0.102
MST-AI MST scale Memberships	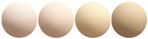 1,2,3,4 0.133, 0.129, 0.118, 0.117	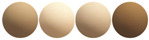 5,3,4,6 0.130, 0.127, 0.125, 0.121	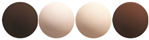 8,1,2,7 0.149, 0.139, 0.120, 0.117

**Table 5:** Comparison of results for the three methods using three performance metrics (@k: top-k).

Metric	Image K-Means				Skin K-Means				MST-AI			
	@1	@2	@3	@4	@1	@2	@3	@4	@1	@2	@3	@4
Kendall’s Tau	-	0.12	0.12	−0.02	-	0.12	0.17	−0.05	-	0.78	0.73	0.74
Spearman’s Correlation	-	0.12	0.13	−0.06	-	0.12	0.18	−0.10	-	0.78	0.73	0.74
NDCG	0.89	0.90	0.91	0.92	0.91	0.93	0.94	0.94	0.99	0.99	0.99	1.00

**Table 6:** *p*-values for two-tailed Student’s *t*-test between methods; dashed lines between methods indicate a statistically significant difference in their results, whereas solid lines represent differences that are not statistically significant.

Top-k	Correlations	NDCG
top-1	N/A	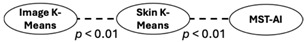
top-2	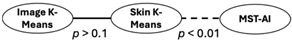	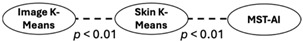
top-3	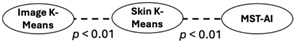	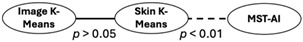
top-4	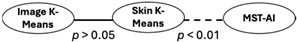	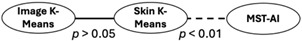

## Data Availability

The ISIC data is publicly available at https://challenge.isic-archive.com/ (accessed on 27 May 2025). Skin Color Dataset, which is annotated by our team, is also publicly available at https://www.kaggle.com/datasets/vahidkermani/skin-color-in-isic/data (accessed on 27 May 2025).

## Abbreviations

The following abbreviations are used in this manuscript:

AI	Artificial Intelligence
ISIC	International Skin Imaging Collaboration
MST	Monk Skin Tone
GMM	Gaussian Mixture Model
VB-GMM	Variational Bayesian Gaussian Mixture Model
KLD	Kullback-Leibler Divergence
DDI	Stanford Diverse Dermatology Images
FST	Fitzpatrick Skin Types
ROI	Region of Interest
PDF	Probability Distribution Function
RGB	Red, Green, Blue
NDCG	Normalized Discounted Cumulative Gain

## Appendix A

Computational Considerations for Distance Measurements

Kullback-Leibler Divergence (KLD) on a Gaussian Mixture Model (GMM) can easily cause under/overflow. Here, we attempt to modify the equations to efficiently compute the KLD of the GMM without computational errors. Starting with the KLD definition:

(A1) $$D_{KL}\left(P∥Q\right)=\sum_{x\in \chi }P\left(x\right)\log\left(\frac{P\left(x\right)}{Q\left(x\right)}\right)$$

where $P\left(x\right)$ and $Q\left(x\right)$ are two PDFs.

Since color distributions are modeled with VB-GMM, their distribution is a Gaussian mixture and requires exponential computation.

(A2) $$P\left(x\right),Q\left(x\right)\sim exponential\;of{-(x-\mu )}^{2}$$

when *x* is close to one of the means of P and far from any means of Q, then P is exponentially larger than Q, and the fraction of $\frac{P}{Q}$ does not fit in the largest conventional floating-point storage, such as 64-bit and 128-bit floating-point. The use of unconventional floating-point containers also adds significant computational overhead and should be prohibited in computationally expensive applications.

(A3) $$P\ll Q⟹\log\left(\frac{P}{Q}\right)\to -\infty \;P\gg Q⟹\log\left(\frac{P}{Q}\right)\to +\infty \;D_{KL}\left(P∥Q\right)=\sum_{x\in \chi }P\left(x\right)\log\left(\frac{P\left(x\right)}{Q\left(x\right)}\right)\to NaN$$

This computational error is an indirect impact of the exponential terms in the GMM. However, if we insert the exponential terms in the computation, we can solve this issue as follows.

Assume that

(A4) $$P\left(x\right)=\frac{1}{s_{p}}\exp\left(x_{p}\right),\;Q\left(x\right)=\frac{1}{s_{q}}exp(x_{q})$$

where $x_{p}$ and $x_{q}$ are GMM logarithmic scores of P and Q at a single point of x, and $s_{p}$ and $s_{q}$ are the summations of samples of distributions P and Q, respectively. Based on these definitions, we can embed P and Q in KLD, expand and simplify as

(A5) $$\begin{array}{cc} D_{KL}\left(P∥Q\right) & =\sum_{x\in \chi }P\left(x\right)\log\left(\frac{P\left(x\right)}{Q\left(x\right)}\right)\\ & =\sum_{x\in \chi }\frac{1}{s_{p}}\exp\left(s_{p}\right)\log\left(\frac{\frac{1}{s_{p}}\exp\left(x_{p}\right)}{\frac{1}{s_{q}}\exp\left(x_{q}\right)}\right)\\ & =\sum_{x\in \chi }\frac{1}{s_{p}}\exp\left(x_{p}\right)\left(\log\left(\frac{1}{s_{p}}\exp\left(x_{p}\right)\right)-\log\left(\frac{1}{s_{q}}\exp\left(x_{q}\right)\right)\right)\\ & =\sum_{x\in \chi }\frac{1}{s_{p}}\exp\left(x_{p}\right)\left(\log\left(\frac{1}{s_{p}}\right)+\log\left(\exp\left(x_{p}\right)\right)-\log\left(\frac{1}{s_{q}}\right)-\log\left(\exp\left(x_{q}\right)\right)\right)\\ & =\sum_{x\in \chi }\frac{1}{s_{p}}\exp\left(x_{p}\right)\left(x_{p}-\log s_{p}-x_{q}+\log s_{q}\right) \end{array}$$

We must notice that $x_{p}$ and $x_{q}$ are functions of x.

The final equation is unlikely to underflow or overflow as long as the input variables fall within the range of float32 or float64.
